# Association of Adverse Childhood Experiences With Health-Related Quality of Life and Quality-Adjusted Life-Years Among Adolescents in the United Kingdom: Findings From Two General Population Cohorts

**DOI:** 10.31083/AP49284

**Published:** 2026-06-29

**Authors:** Amarech Obse, Kamaldeep Bhui, Laura Havers, Sania Shakoor, Georgina Hosang, Siobhan Hugh-Jones, Minhua Ma, Anna Mankee-Williams, Alexis Karamanos, Seeromanie Harding, Paul McCrone

**Affiliations:** ^1^The Institute for Lifecourse Development, University of Greenwich, Old Royal Naval College, Park Row, SE10 9LS London, UK; ^2^Department of Psychiatry, Nuffield Department of Primary Care Health Sciences, and Wadham College, University of Oxford, OX3 7JX Oxford, UK; ^3^Oxford Health, Oxford and East London NHS Foundation Trusts, OX4 4XN London, UK; ^4^World Psychiatric Association Collaborating Centre, OX3 7JX Oxford, UK; ^5^Centre for Psychiatry and Mental Health, Wolfson Institute of Population Health, Queen Mary, University of London, E1 4NS London, UK; ^6^School of Psychology, University of Leeds, LS2 9JT Leeds, UK; ^7^Department of Psychiatry, University of Oxford, OX3 7JX Oxford, UK; ^8^Research and Innovation Department, Falmouth University, TR10 9FE Falmouth, UK; ^9^Department of Population Health Sciences, School of Life Course & Population Sciences, Faculty of Life Sciences and Medicine, King’s College London, SE1 1UL London, UK

**Keywords:** adolescent, adverse childhood experiences, health-related quality of life, quality-adjusted life-year, United Kingdom

## Abstract

**Background:**

Evidence on the impact of adverse childhood experiences (ACEs) on multidimensional wellbeing, including health-related quality of life (HRQoL) and quality-adjusted life-years (QALYs), remains inadequate, particularly among young people. This study examines how ACEs and their interactions with sex, ethnicity, and socioeconomic status (SES) are associated with HRQoL and QALYs in United Kingdom (UK) adolescents.

**Methods:**

Baseline and follow-up data from the Determinants of Adolescent Social Well-being and Health (DASH) Study (N = 6648) and HeadStart Cornwall (HeadStart) (N = 4575) were used. ACE data were sourced from self-reports and official records and measured as binary and count variables. The Strengths and Difficulties Questionnaire (SDQ) was mapped to the Child Health Utility 9D (CHU9D) to calculate utility values for QALY estimation.

**Results:**

Over the 3- and 2-year follow-up periods, mean accumulated QALYs were 2.30 (standard error [SE]: 0.002) (DASH) and 1.45 (SE: 0.004) (HeadStart). The occurrence of any ACEs, self-reported and officially recorded, respectively, reduced QALYs by 0.064 (SE: 0.021) (DASH) and 0.060 (SE: 0.021) (HeadStart). These translate to losses of 23 (DASH) and 22 (HeadStart) days over the follow-up period. A greater count of ACEs was associated with stronger effects. Dose-dependent interactions were significant only for ACEs-ethnicity and ACEs-sex (≥2 ACEs), with White adolescents and females experiencing greater QALY losses as the number of ACEs increased.

**Conclusions:**

ACEs, whether self-reported or officially recorded, were associated with greater QALY losses, with the strength of association increasing as the number of ACEs increased. Furthermore, White and female adolescents exposed to a greater number of ACEs had the highest QALY losses. Further studies are needed to investigate the moderation of these associations and the mechanisms underlying the associations between ACEs and HRQoL/QALYs to inform targeted interventions.

## Main Points

1. Adverse childhood experiences (ACEs) were associated with lower health-related quality of life (HRQoL) in adolescents.

2. Female adolescents had significantly lower quality-adjusted life years (QALYs) than males.

3. An increasing number of ACEs was linked to a graded reduction in HRQoL.

4. There was a dose-dependent interaction effect of ACEs and gender on QALYs.

## 1. Introduction

Adverse childhood experiences (ACEs) are stressful, often traumatic events occurring during childhood. These events have negative physical and psychological effects that can potentially last throughout the life course [1]. A landmark study on ACEs identified three groups of adversity occurring before the age of 18 that are linked with negative health consequences: abuse (physical, sexual, emotional), neglect (physical, emotional), and family dysfunction (divorce/separation, mental illness, substance misuse, domestic violence, and family member incarceration) [1]. A wealth of subsequent studies has corroborated these findings in different contexts [2]. An expanded definition of ACEs includes events experienced at a community level, such as witnessing violence, discrimination, unsafe neighbourhoods, bullying, and being placed in foster care, which have been shown to have similar effects on health and wellbeing [3].

Several large-scale reviews provide strong evidence for the disturbingly high global prevalence rates of ACEs. One meta-analysis of 244 publications from across the world estimated the prevalence of self-reported ACEs at 12.7% for sexual abuse (18% among girls and 7.6% among boys), 22.6% for physical abuse, and 36.3% for emotional abuse, with lower rates for ACEs recorded in official reports (0.4% for sexual abuse, 0.3% for physical abuse, and 0.3% for emotional abuse) [4]. A more recent systematic review of 337 global studies estimated higher rates of prevalence, with a median lifetime prevalence of sexual abuse among girls in Africa of 18.9%, in North America of 20.4%, and in Australia of 28.8%, with lower rates for boys [5]. Physical abuse rates were highest in African girls (50.8%) and boys (60.2%) and lowest in Europe, with 12% for girls and 27% for boys. Furthermore, the review revealed considerable variation in self-reported lifetime prevalence of ACEs among studies from the UK for physical abuse (3.6% to 32.6%), sexual abuse (0.7% to 27.8%), and emotional abuse (4% to 66.7%). Data on the prevalence of neglect is usually sparse, with substantial variation between studies [4,5]. Moody et al. [5] found that the prevalence of neglect varied between 5.6% to 77.8% in the UK, based on a small number of studies.

Numerous studies have shown that ACEs are strongly linked with objective measures of physical and mental health, as well as behavioural problems [1,2,6]. For instance, Felitti et al. [1] showed that individuals with four or more ACEs were more than twice as likely to have ischemic heart disease (adjusted odds ratio [AOR] = 2.2, 95% CI: 1.3–3.7) and stroke (AOR = 2.4, 95% CI: 1.3–4.3) than those with no ACEs. Similarly, the odds of suicide attempt and problematic drug use were 30.14 (14.73–61.67) and 10.22 (7.62–13.71) times higher in individuals with four or more ACEs compared to those with no ACEs, as estimated through a meta-analysis [2]. Additionally, unpredictable early life circumstances, such as unstable family environment and inconsistent parenting, were associated with a higher likelihood of non-suicidal self-injury [7]. Dose-dependent evidence further indicates that adolescents under 21 years of age exhibit higher rates of non-suicidal self-injury with an increasing number of ACEs [8].

Despite the well-documented links between ACEs and objective measures of health, little is known about how the effects of ACEs extend to subjective, self-rated health, reflecting individuals’ own perspectives of their physical, mental, emotional, and social functioning. Subjective measures are considered an important complement to objective indicators of health, providing a more holistic view of health [9]. This is important because self-rated health is a significant predictor of mortality and morbidity, adjusting for objective measures of health [10]. Furthermore, improvements in objective measures of health do not always correspond to better day-to-day functioning, or vice versa [9]. Hence, it is recommended that care, research, and policy incorporate both objective and subjective measures of health [9,10,11].

An important subjective outcome measure used in health and social care research and economic evaluations is health-related quality of life (HRQoL). HRQoL is a preference-based, multidimensional (e.g., physical, psychological, social) measure of well-being that captures social functioning, role limitations, subjective evaluation of general health, and life satisfaction [12]. In economic studies, HRQoL is often treated as a proxy for ‘utility’. Utility values for various health states are estimated from target populations’ preferences using direct methods (e.g., standard gamble and time trade-off) or multi-attribute health status classification systems (e.g., EQ-5D-5L, SF-6D) [12,13,14]. The main use of utility values in economic evaluations is to construct quality-adjusted life years (QALYs). QALY is a summary measure of ‘quality’ and ‘quantity’ of life computed by weighting the length of life by utility values [12]. QALYs enable policymakers to compare across diverse healthcare areas. In England and Wales, the National Institute for Health and Care Excellence (NICE) values a QALY at between £20,000 (US $27,000) and £30,000 (US $40,500) [15]. However, the UK Treasury values a QALY at £60,000 (US $81,000) [16].

When data on preference-based measures of health are unavailable in a study, non-preference-based measures can also be used to predict generic preference-based health-state utility values [17]. While the methods for obtaining health utilities for adults are well established, those for children and adolescents are still under development [18,19,20]. HRQoL in children is either self-rated or proxy rated (e.g., by parents) [21,22]. Validated generic preference-based multi-attribute health status classification systems for children and adolescents include Child Health Utility 9D (CHU9D), EQ-5D-Y, and Quality of Well-Being scale (QWB) [23]. When preference-based measures of quality of life have not been used in a particular study, a common approach is to map from other measures onto a preference-based measure when both have been used in a separate study [17].

Previous studies have investigated the association between ACEs and HRQoL in adults and adolescents [24,25,26] as well as other indicators of life satisfaction [27]. A systematic review showed an inverse relationship between child maltreatment and HRQoL among children and adult survivors [26]. Other recent studies report similar associations [24,25,28,29]. For instance, among adolescents aged 11 to 20, HRQoL, as measured by the Paediatric Quality of Life Inventory, was lower among those reporting ACEs [30,31]. Furthermore, HRQoL progressively and significantly decreased with increasing number of ACEs, with reductions of 2.99 (one ACE), 6.05 (two ACEs), and 14.7 (three or more ACEs) units relative to no ACEs [31]. Additionally, Lo et al. [29] showed that poorer physical and emotional functioning in adolescents was specifically associated with psychological aggression and neglect, respectively. In contrast, impaired social functioning was affected by all three types of adversity: physical abuse, psychological abuse and neglect. A study that estimated quality-adjusted life expectancy (QALE) by weighting expected life years by HRQoL found that QALE decreased with increasing ACEs [32]. For an 18-year-old individual with no ACEs, QALE was estimated at 55.1 years, compared to 45.6 years for those with three or more ACEs [32]. Furthermore, ACEs were associated with lower positive functioning — happiness, optimism, connectedness, and perseverance — among adolescents [27].

Multiple mechanisms explain the relationship between ACEs and poor HRQoL, such as pathways involving poor mental health, behavioural risk, biological stress, and impaired sleep and functioning [2,33,34,35]. ACEs primarily affect HRQoL by influencing health-risk behaviours and chronic diseases [1,2], both of which contribute to reduced HRQoL. Exposure to four or more ACEs is particularly associated with greater odds of poor mental health, chronic diseases, smoking, and alcohol misuse, which are the main drivers of disability and reduced quality of life [1,2,36]. Chronic stress due to ACEs was also reported to have effects on neurobiological stress mechanisms, resulting in impairment of brain structure and function [33]. Additionally, adolescents with ACEs have sleep problems, which are partially mediated by depression and anxiety, which further contribute to reduced HRQoL [35].

In summary, the available research shows that ACEs are associated with poorer subjective health, with limited evidence on how these associations vary by the youth’s sociodemographic characteristics. Using two UK cohort datasets, this study aims to assess the association of ACEs with HRQoL and QALYs. It also investigates the differential association of ACEs with HRQoL and QALYs by sex, ethnicity, and socioeconomic status (SES) of study participants, for which evidence is limited. The datasets used for the analyses differ in their sources of ACEs: self-report versus government-recorded. Different sources of ACEs data capture different subsets of adversity and introduce their own biases [37]. Usually, official records capture the most severe cases that come to official attention, whereas self-reports indicate broader subjective ACEs [37]. Recall bias in retrospective ACE reporting is associated with both under- and over-reporting [38,39], whereas official records often underestimate the prevalence of ACEs [4]. Various factors contribute to non-agreement between retrospective and prospective ACEs, such as intentional withholding or fabrication of information or measurement issues [37]. Therefore, populations with ACE self-reports and administrative data may have different experiences and risk mechanisms for the outcomes of interest [37,40].

Accordingly, the populations with ACE exposure in the two datasets used for this study are not directly comparable. Therefore, the study presents complementary analyses, though it answers the same questions to determine whether legally documented ACEs and broader self-reported ACE exposure show comparable associations with HRQoL/QALY. The use of the two datasets — with different sources of ACE data, covering different geographic areas, time periods, and ethnic composition of study participants — allows triangulation of findings, strengthening the evidence validity and generalisability.

## 2. Materials and Methods

Data were from two UK cohort studies: the Determinant of Adolescent Social well-being and Health (DASH) Study [41], and HeadStart Cornwall (HeadStart) [42]. The DASH survey was a longitudinal adolescent cohort study, with sampling concentrated in London boroughs with high ethnic minority populations [41,43]. It was conducted in three waves in 51 schools from 10 London boroughs. At baseline, 6648 students aged 11–13 years completed the survey in 2002/03. The first follow-up survey was conducted in 2005/06, during which 4785 students were retained, the focus of the current study [41]. The National Health Service Southeast Scotland Research Ethics Committee approved the study. Parents were informed about the study and given the option to have their child opt out. All participating adolescents also provided informed consent or assent prior to completing the questionnaires [43].

HeadStart was a cross-sectional and longitudinal survey of approximately 12,000 adolescents aged 11–16 years attending all state schools in Cornwall between 2017–2021 (National Evaluation of HeadStart: Headstart Kernow). For this study, we used the longitudinal data from waves 2017 and 2019 (age 13–14), with a baseline sample of 4575 Year 7 pupils. The baseline sample comprised 3786 respondents, of whom 2705 were retained in the 2019 follow-up. Cornwall consists of 96.8% of the population who identify as “White” in 2021 [44]. Ethical approval for HeadStart study was granted by the University College London Ethics Committee. Pupil assent and parental consent for HeadStart participation were obtained before data collection [45].

### 2.1 Measures

The dependent variables used in the analyses are HRQoL and QALYs. Neither DASH nor HeadStart used a preference-based utility measure. However, the studies included the self-report Strengths and Difficulties Questionnaire (SDQ) [46] administered at baseline and follow-up.

The SDQ is a behaviour and mental health screening and outcome questionnaire consisting of 25 questions with three-option responses: 1 (not true), 2 (somewhat true), and 3 (certainly true). Higher values in each subscale indicate poorer functioning [47]. Utility values from the SDQ were derived via mapping onto the Child Health Utility 9D (CHU9D) measure [48,49]. The CHU9D is a multi-attribute utility instrument with nine domains (worried, sad, tired, annoyed, homework/schoolwork, sleep, daily routine, and activities) developed to estimate health-state utilities in children and adolescents [48]. The mapping algorithm was estimated using a generalized linear model (GLM) with a power link (λ = 2) [49]. The utility values range from 1 (full health) to 0 (death). While HRQoL solely measures the quality of life, QALYs combine the ‘quality’ and ‘quantity’ of life into a single index. In QALYs, a year lived with perfect health is assumed to be worth one QALY, and a year of life lived with less than perfect health is assumed to be worth less than one QALY [13]. QALYs are estimated using an ‘area under the curve’ (AUC) approach, assuming linear change over time [50]. The average utility values at baseline and follow-up are weighted by duration of follow-up, specifically 3 years for DASH and 2 years for HeadStart.

ACE is the main independent variable of interest. However, there is a considerable difference in the measurement of ACEs in the two datasets. DASH includes various measures of self-reported ACEs: being placed in foster care, parental divorce/separation, parental death, parental mental health problem or cancer and ‘hassle’ endured by the respondents due to race, language, and/or looks (**Supplementary Table 1**). These variables were binary, indicating the occurrence (or non-occurrence) of the adversities. A count variable indicating the number of ACEs experienced was created by summing the raw counts of these adversities. Furthermore, a binary variable indicating the presence of the ACEs was generated.

The HeadStart data include one variable indicating whether ACEs are present (‘on family list’). Being on the family list indicates whether the respondent’s family (the child or the parent) was receiving support from local government through the Supporting Families Program [51]. Families are listed on this program if they experience adversities due to sexual exploitation, crime, homelessness, domestic violence, unemployment, or physical and/or mental illness in the household. Data on the types of adversities experienced are not available. Because the two datasets measure distinct constructs of ACEs — retrospective self-report and prospective official records — analyses and findings are presented separately. This approach facilitates the assessment of consistency or differences in associations across ACE sources.

Other characteristics of interest are sex, ethnicity, and socioeconomic status (SES). In HeadStart data, SES was indicated by eligibility for free school meals (FSM) [52]. Based on the FSM criteria, the respondents were categorised as poor (10.07%) or non-poor (89.93%) in 2017. In the DASH data, household asset ownership (**Supplementary Table 2**) was used to generate an asset index using multiple correspondence analysis (MCA) [53], which was then used to measure SES. For a comparative analysis, respondents in the lowest 10% of the asset index (firststdecile) were categorised as poor, and the rest as non-poor. Additionally, a continuous asset index was used in a separate analysis. HeadStart includes a binary ethnicity variable (White vs non-White). To maintain comparability across datasets and simplify analyses, ethnicity in the DASH data was also grouped in the same way, even though more detailed ethnic classifications were originally available in DASH.

### 2.2 Data Analysis

DASH and HeadStart data were analysed separately. Descriptive statistics is reported for the observed data using frequencies (percentages) for categorical variables and means (standard deviation (SD)) for quantitative variables.

Multivariable regression of the follow-up total SDQ score showed that dropout was highly significantly associated with baseline total SDQ score, gender, and race/ethnicity (*p* = 0.000), whereas self-reported ACEs and SES did not predict dropout in the DASH data. Respondents with higher SDQ scores, females, and White participants were more likely to drop out. In contrast, being on the Family List — a record of ACEs from government administrative data — was the only significant predictor of dropout in HeadStart data (**Supplementary Table 3**). These findings suggest that the Missing At Random (MAR) assumption is plausible. Therefore, these covariates were included when imputing missing data in the datasets. Missing data were imputed using Multiple Imputation by Chained Equations (MICE) in Stata 18 [54,55,56]. 50 imputed datasets (with 10 iterations per imputation) were generated for each dataset. The imputation method was determined based on the measurement type of each variable (i.e., predictive mean matching for continuous and count data and logistic for binary variables). The number of missing values imputed for each variable is reported in the (**Supplementary Table 4**). Imputation convergence and the plausibility of imputed data were assessed by examining Monte Carlo errors [56] and comparing the observed and imputed data. Monte Carlo errors for the estimated coefficients were considered acceptable if they were less than 10% of the standard errors of the coefficients, indicating an adequate number of imputations [56].

Utility scores and QALYs were generated using the mapping algorithm [49] with passive imputation within each imputed dataset. The predictive performance of the mapping algorithm was assessed across multiple imputed datasets through visual inspection of histograms of predicted utilities, analyses of summary statistics, and examination of ceiling and floor effects. Furthermore, the association between the predicted utilities and total SDQ was examined using bivariate analysis. The final analysis models were also estimated using total SDQ as a dependent variable for triangulation. All analyses were conducted separately on each imputed dataset, and the results were pooled according to Rubin’s method [57]. For comparative analysis, ACEs were included as dummy variables in each dataset. Further analyses of the graded effect of ACEs were conducted using a multi-category ACE variable (none, one, two, three or more) in the DASH data. Interaction terms were included in all models for both datasets, specifically for (i) ACEs × SES and (ii) ACEs × sex. An additional ACEs × ethnicity interaction was examined only in the DASH dataset, as the HeadStart sample contained limited ethnic variation (only 2.38% identified as non-White). The supplementary DASH analyses also incorporated a continuous asset index measure.

### 2.3 Sensitivity Analysis

Sensitivity analysis was conducted using inverse probability weighting (IPW) to assess the robustness of the association between ACEs and HRQoL and QALYs to potential confounding [58,59]. IPW analysis was conducted within the imputed datasets. Propensity scores indicating the probability of exposure to ACEs were estimated, adjusting for gender, race/ethnicity, and SES. Inverse probability weights were then used to estimate the average treatment effect on the treated (ATET) among ACE-exposed individuals. ATET shows how much outcomes differ for individuals exposed to ACEs compared with what their outcome would likely have been if they were not exposed to ACEs. IPW-weighted regression models were estimated within each imputed dataset, and estimates were pooled across imputations using Rubin’s rules [57]. Because the IPW analysis aimed to assess the robustness of the overall associations to confounding, interaction terms were not included in the sensitivity analyses models.

## 3. Results

### 3.1 Descriptive Statistics

The average age of the respondents was 12.60 (SD: 0.62) (DASH) and 11–12 years (HeadStart). The two surveys had a similar sex composition, both with a slightly higher proportion of male respondents. Ethnic composition differed significantly: 29.59% White in DASH versus 97.62% in HeadStart. The proportion of DASH respondents with self-reported ACEs (59.23%) was much higher compared to the official record of ACEs in HeadStart (12.44%). Average HRQoL reduced between baseline and follow-up surveys in both datasets. On average, respondents accumulated 2.33 (SE: 0.002) (DASH) and 1.45 (SE: 0.004) (HeadStart) QALYs over the 3-year and 2-year follow-up periods, respectively, which indicate losses of 0.67 (DASH) and 0.55 (HeadStart) QALYs (Table 1)

**Table 1.** **Demographic characteristics DASH (2002/03) and HeadStart (2017) respondents at baseline**.

			**DASH**		**HeadStart**	
			**N/Mean**	**%/Std. dev**	**N/Mean**	**%/Std. dev**
Age (mean, SD)^a^			12.600	0.620		
**Sex**						
	Male		3535	53.350	2168	51.040
	Female		3091	46.650	2080	48.960
**Ethnicity**						
	White		1962	29.590	4110	97.620
	Non-White		4669	70.410	100	2.380
**ACEs**						
	Administrative data					
		Has official record of ACEs			531	12.440
		No official record of ACEs			3738	87.560
	Self-report					
		One or more ACEs	3718	59.230		
		No ACEs	2559	40.770		
		One ACE	2149	34.230		
		Two ACEs	1031	16.430		
		Three or more ACEs	538	8.570		
		ACE count (mean, Std. Dev)	0.949	1.011		
**Socioeconomic status**						
	From non-poor households		5612	89.99	3839	89.930
	From poor households		624	10.01	430	10.070
**HRQoL / QALYs **(mean^b^, SE)						
	Baseline HRQoL		0.791	0.001	0.743	0.002
	Follow-up HRQoL		0.746	0.001	0.712	0.002
	QALYs		2.330	0.002	1.450	0.004

^a^There was no age data for HeadStart survey respondents. Respondents were Year 7 pupils with an average age of 11–12. ^b^Pooled mean estimate after multiple imputation. ACEs, adverse childhood experiences; HRQoL, health-related quality of life; QALYs, quality-adjusted life years.

### 3.2 Robustness Analyses

The robustness assessment of the multiple imputation indicated no evidence of non-convergence, as coefficients remained stable across iterations and Monte Carlo errors of the estimates were less than 10% their corresponding standard errors [56] (**Supplementary Table 5**). Similarly, results of the robustness analysis of the mapping algorithm showed that predicted utilities vary within the feasible range of 0 and 1 and displayed a left-skewed distribution, as expected, with consistent ceiling (>0.95) and floor (<0.3) effects (**Supplementary Fig. 1**). The pooled mean utilities at baseline and follow-up were, respectively, 0.791 (SE: 0.001) and 0.746 (SE: 0.001) in DASH and 0.743 (SE: 0.001) and 0.711 (SE: 0.002) in HeadStart. These estimates were comparable with those (0.798; SD: 0.12) reported by Sharma et al. [49]. The mean utility values were also consistent with estimates of utility in the UK child and adolescent population [60]. Furthermore, bivariate regressions showed a highly significant negative association between the predicted utilities and total SDQ in both datasets. Mean utilities decreased monotonically as the severity of SDQ increased (**Supplementary Tables 6–8**). These results of robustness assessment suggest that the mapping of the SDQ to CHU9D was plausible.

### 3.3 Association of Occurrence of ACEs With HRQoL and QALYs

Overall, the occurrence of ACEs — exposure to any ACEs compared to none — predicted lower HRQoL as measured by both self-reported and official records of ACEs, adjusting for sex, SES, and ethnicity (Table 2). On average, the HRQoL of DASH respondents who self-reported ACEs was 0.031 (SE = 0.009) lower than that of their peers without ACEs at baseline, and 0.030 (SE = 0.009) lower at follow-up. Reductions in HRQoL were slightly higher at baseline, using the official ACE record (–0.046, SE = 0.012). Females had significantly impaired QALYs and lower HRQoL in both surveys. Ethnicity significantly predicted HRQoL and QALY in DASH data but not in HeadStart data. QALYs were on average –0.064 (SE = 0.021) and –0.060 (SE = 0.021) lower, respectively, using self-report and official records of ACEs in reference to no ACEs. This is equivalent to losses of about 23 (DASH) and 22 (HeadStart) days over the three- and two-year follow-up period. The interactions between ACEs and sex resulted in inconsistent estimates on utilities but a significant reduction of QALYs in females compared to males. The interaction between ACEs and SES was insignificant in either dataset.

**Table 2.** **Multiple imputation estimates of predictors of health-related quality of life (HRQoL) and baseline predictors of quality-adjusted life-years (QALYs) in DASH and HeadStart respondents**.

			HRQoL				QALYs	
			DASH		HeadStart		DASH	HeadStart
			2002/03	2004/2005	2017	2019	2002/03–2005/06	2017–2019
			Coefficient (SE)	Coefficient (SE)	Coefficient (SE)	Coefficient (SE)	Coefficient (SE)	Coefficient (SE)
Main effects								
	ACEs							
		Administrative data			–0.046 (0.012)***	–0.015 (0.014)		–0.060 (0.021)***
		Self-report	–0.031 (0.009)***	–0.030 (0.009)***			–0.064 (0.021)***	
	Female		–0.036 (0.004)***	–0.075 (0.004)***	–0.041 (0.004)***	–0.093 (0.005)***	–0.162 (0.010)***	–0.133 (0.008)***
	Non poor		0.014 (0.007)**	0.009 (0.008)	0.019 (0.007)***	0.031 (0.009)***	0.042 (0.018)**	0.051 (0.013)***
	White		–0.012 (0.003)***	–0.010 (0.003)***	–0.009 (0.011)	–0.021 (0.013)	–0.030 (0.007)***	–0.029 (0.020)
Interactions								
	Female and ACEs		–0.014 (0.005)***	0.002 (0.005)	–0.022 (0.011)*	–0.038 (0.014)***	–0.028 (0.013)**	–0.060 (0.021)***
	Non poor and ACEs		0.004 (0.009)	0.005 (0.100)	0.006 (0.013)	–0.025 (0.015)	0.003 (0.022)	–0.019 (0.023)
	Constant		0.819 (0.007)***	0.790 (0.008)***	0.763 (0.013)***	0.757 (0.015)***	2.400 (0.017)***	1.520 (0.024)***
	Number of imputations		50		50		50	50
	Number of observations		6648		4575		6648	4575

***, **, and * indicate significance at *p* < 0.01, *p* < 0.05, and *p* < 0.1, respectively. Unstandardised coefficients. The coefficient estimates of QALYs represent the total cumulative change in QALYs over the entire follow-up period—3 years for DASH and 2 years for HeadStart.

### 3.4 Graded Association of ACEs With HRQoL and QALYs

HRQoL and QALYs declined significantly with increasing ACE counts, indicating a dose-dependent (graded) effect (Table 3). On average, baseline HRQoL was –0.011 (SE = 0.004), –0.031 (SE = 0.006), and –0.063 (SE = 0.007) in respondents with one, two, and three or more ACEs, respectively, compared to those with no ACEs. Over the follow-up period, respondents with two (–0.067, SE = 0.032) and three or more (–0.149, SE = 0.036) ACEs accrued fewer QALYs, on average, compared to the non-exposed respondents. These translate to losses of 24 days and 54 days over the three-year follow-up period, respectively. Additionally, ACEs had a greater effect on HRQoL (baseline) and QALYs among White adolescents, with the magnitude of association increasing with a greater number of exposures. A dose-dependent interaction of ACEs and gender was observed at higher levels of ACEs (2+ ACEs). The dose-dependent interactions between ACEs and SES were inconclusive.

**Table 3.** **Multiple imputation analysis of dose-dependent relationships between ACEs and HRQoL and QALYs in the DASH sample**.

			HRQoL 2002/03	HRQoL 2004/05	QALYs
			Coeff. (SE)	Coeff. (SE)	Coeff. (SE)
Main effects					
	Self-reported number of ACEs (ref. none)				
		One	–0.011 (0.004)**	–0.018 (0.004)***	–0.015 (0.025)
		Two	–0.031 (0.006)***	–0.034 (0.006)***	–0.067 (0.032)**
		Three or more	–0.063 (0.007)***	–0.057 (0.009)***	–0.149 (0.036)***
	Female		–0.036 (0.004)***	–0.075 (0.004)***	–0.161 (0.010)***
	Asset index		0.005 (0.002)***	0.001 (0.002)	0.041 (0.003)**
	White		–0.006 (0.004)	–0.011 (0.004)***	–0.011 (0.011)
Interactions					
	*Ethnicity and ACEs*				
		White and one ACEs	–0.008 (0.006)	0.004 (0.006)	–0.027 (0.015)*
		White and two ACEs	–0.021 (0.008)***	–0.008 (0.009)	–0.047 (0.019)**
		White and three or more ACEs	–0.024 (0.011)**	0.004 (0.015)	–0.064 (0.027)**
	*Asset index and ACEs*				
		Asset index and one ACE	0.001 (0.003)	–0.001 (0.003)	–0.004 (0.024)
		Asset index and two ACEs	–0.005 (0.004)	0.002 (0.003)	–0.009 (0.032)
		Asset index and three or more ACEs	–0.002 (0.004)	–0.007 (0.006)	–0.018 (0.037)
	*Gender and ACEs*				
		Female and one ACE	–0.008 (0.006)	0.004 (0.006)	–0.013 (0.014)
		Female and two ACEs	–0.021 (0.008)***	0.005 (0.008)	–0.038 (0.019)**
		Female and three or more ACEs	–0.022 (0.010)**	–0.016 (0.012)	–0.056 (0.024)**
		Constant	0.830 (0.003)***	0.799 (0.003)***	2.39 (0.018)***
		Number of observations	6648		
		Number of imputations	50		

***, **, and * indicate significance at *p* < 0.01, *p* < 0.05, and *p* < 0.1, respectively. The coefficient estimates of QALYs represent the total cumulative change in QALYs over the entire follow-up period—3 years for DASH and 2 years for HeadStart.

### 3.5 Sensitivity Analyses

The unweighted regression model in DASH data, without interaction terms, showed that self-reported ACE exposure lowered QALYs on average by 0.074 (SE = 0.006). Sensitivity analyses with inverse probability weighting also showed that QALYs were, on average, lower by 0.075 (SE = 0.006) among adolescents who experienced ACEs than they would likely be if they were not exposed to ACEs. Similarly, in the official ACEs record, both unweighted and inverse-probability-weighted regressions for QALY produced nearly identical results. Results of IPW-weighted regressions of baseline and follow-up utilities also showed strong consistency with the unweighted estimates (Table 4). These results suggest that the association between ACEs and HRQoL/QALYs were robust to confounding adjustment.

**Table 4.** **Sensitivity analyses of association between ACEs and QALYs/HRQoL using inverse probability weighting**.

		**DASH (self-reported ACEs)**			**HeadStart (official record of ACEs)**		
		**Effect of ACEs**	**SE**	***p***	**Effect of ACEs**	**SE**	***p***
QALYs							
	Unweighted regression	–0.074	0.006	0.000	–0.101	0.012	0.000
	IPW weighted regression - ATET	–0.075	0.006	0.000	–0.098	0.013	0.000
	Counterfactual mean (no ACEs)	2.346	0.004	0.000	1.452	0.008	0.000
Follow-up utility							
	Unweighted regression	–0.015	0.003	0.000	–0.048	0.008	0.000
	IPW weighted regression - ATET	–0.016	0.003	0.000	–0.045	0.009	0.000
	Counterfactual mean (no ACEs)	0.753	0.002	0.000	0.708	0.005	0.000
Baseline utility							
	Unweighted regression	–0.034	0.003	0.000	–0.053	0.006	0.000
	IPW weighted regression - ATET	–0.035	0.003	0.000	–0.053	0.007	0.000
	Counterfactual mean (no ACEs)	0.811	0.002	0.000	0.744	0.004	0.000

Notes: ATET, average treatment effect on treated.

## 4. Discussion

This study examined the association between ACEs and both HRQoL and QALYs in two UK adolescent cohort surveys that differed in ACE data source, geographic region, time period, and ethnic composition, thereby contributing to growing evidence on how ACEs affect the multidimensional wellbeing of adolescents. It also assessed whether these associations varied by sex, ethnicity, and SES. Both self-report and government-recorded ACEs were associated with lower HRQoL and fewer QALYs, with stronger negative effects as the number of ACEs increased, demonstrating a dose-dependent relationship. Additionally, graded interaction between ACEs and both ethnicity and gender was identified for QALYs.

HRQoL declined between baseline and the follow-up surveys (2–3 years later) in both groups of respondents, regardless of ACEs. However, adolescents with ACE exposures — both from self-report and official records — generally demonstrated lower HRQoL and accrued fewer QALYs compared to adolescents not exposed to ACEs. These findings align with previous evidence synthesised in a systematic review that reported significant negative associations for both self-reported and proxy-rated HRQoL [26]. Furthermore, direct and indirect exposure to violence was found to be associated with HRQoL compared to other adverse life events not involving victimisation [61]. However, disagreement in the association between ACEs and HRQoL, depending on whether HRQoL was self-reported or proxy-rated, was also reported [62,63]. For instance, a study with children and adolescents in Germany found non-significant effects of ACEs on self-rated HRQoL, whereas proxy-rated HRQoL was lower among maltreated children [63]. In contrast, Jud et al. [62] found significantly impaired self-reported HRQoL but not caregiver-rated HRQoL in maltreated children. Discordance between self-rated and proxy-rated HRQoL is common, likely reflecting differences in perceptions of children’s experiences between children and proxies [64]. Therefore, this discordance should be taken into account when interpreting evidence of associations between ACEs and HRQoL.

HRQoL was significantly reduced in females compared to males in the follow-up surveys, suggesting that sex-related differences in HRQoL emerge as adolescents get older. Given respondents’ baseline age (average of 12 years), reduced HRQoL over time may be explained by a continuous decrease in life satisfaction (LS) in adolescents, beginning around 11 years of age [65]. This aligns with the broad literature showing that LS is generally lower in adolescence compared to pre-adolescence and childhood, with adolescence associated with increased emotional and psychological vulnerability, particularly among girls [65,66,67]. The lowest average LS was reported among 15-year-olds in the UK compared with those in 27 European countries, where one in five (male) and almost one in three (female) reported low LS [68].

Although the evidence of gender or sex differences in LS and social inequality in LS remains inconsistent [67,69], numerous studies show that females generally report lower quality of life [70,71,72]. Notably, the LS gap between adolescent boys and girls is larger in countries with greater gender equality — a phenomenon referred to as the gender-equality paradox [73,74]. Several empirical studies attribute lower LS in adolescent girls to biological, psychological, emotional, and social factors [75,76,77], including earlier maturation, heightened sensitivity to stress and hormonal changes [78], increased levels of negative emotions [79], and reduced self-esteem [80]. For instance, early puberty in girls is associated with stress and body dissatisfaction [81], which is exacerbated by strong societal and media pressures of standards of beauty, body image, and appearance, which lowers the self-esteem of girls [82]. Additionally, cultural and structural inequalities heighten concerns about safety and security among girls, restricting their participation in public spaces and limiting their opportunities [83,84]. Girls are also expected to conform to gender norms and role expectations, which further contribute to stress among girls [85,86].

Other studies, while acknowledging some of these factors, argue that some of these explanations do not account for variations in findings across countries and the gender-equality paradox. These studies argue that broader social and contextual factors, including macroeconomic conditions, play a more significant role in explaining the gender gap in LS [74,87,88]. For example, international studies [74,88,89] found that LS is lower among girls, with the difference greater in more gender-equal countries. Brisson et al. [87] demonstrated that the effect of gender on LS is confounded by other factors influencing LS, and that this effect diminished when these confounders were accounted for. After adjusting for confounders, LS was found to be higher among girls in Kazakhstan, contrary to the result in Luxembourg [73], where LS was higher in boys. Similarly, Eriksson and Strimling [88] reported that 40% of the gender effect on LS was mediated by countries’ gender equality indices. Although the evidence on the gender-equality paradox is still evolving, plausible explanations for the paradox include increased awareness of discrimination against females and greater gender comparisons in countries with greater gender equality, which undermine the progress in gender equality and contribute to lower LS [74].

SES is another household-level determinant of HRQoL and QALYs among adolescents; however, the results should be interpreted cautiously due to measurement issues. First, the measurement of SES in the two datasets varies and is primarily based on financial or material resources. In DASH data, SES was measured using an asset index generated from household assets (e.g., cars, TVs, etc.). Asset indices as measures of SES have limitations, such as the inability to capture current income or consumption and limited comparability due to variation in asset holdings [90,91]. In HeadStart, SES was measured based on eligibility for free school meals (FSM). The eligibility criteria for FSM include, among others, household income below a threshold, receipt of income support, employment support, or other government allowances (Free meals in further education funded institutions guide: academic year 2025 to 2026 - GOV.UK). Hence, the SES index in this study is a partial measure of SES. In fact, while ‘socioeconomic status’ and ‘socioeconomic position’ (SEP) are used interchangeably [92], SEP was suggested as a better term indicating resource-based (e.g. income, education) and prestige-based (status in a social hierarchy) status that are measured at individual, household, and neighbourhood levels, such as education, occupational prestige, housing, neighbourhood deprivation [93]. However, measurement of SES remains inconsistent across studies and is limited to a few or a single indicator [94]. Second, the binary SES indicator in this study does not fully capture the full gradient, with 10% categorised as poor and 90% as non-poor. This limits statistical power and precision of regression estimates [95].

Taking the foregoing into account, the HeadStart data showed a strong association between exposure to ACEs and poorer HRQoL and QALYs, whereas the effect of SES in the DASH data was inconsistent. This may partly reflect differences in how SES was measured, as eligibility for FSM is based on means testing and therefore captures current SES more accurately than asset ownership in DASH, which may not fully represent current SES. Previous studies [62,63] also showed that adolescents from low SES households have reduced HRQoL and QALYs compared to those from high SES households. A multi-country study across seven European countries showed a stable SES gradient in adolescent HRQoL (using parental education and material wealth), with material wealth being more important during adolescence [96]. Another study estimated that lower quality of life was 1.12 times higher in the poorest quintiles (as measured by asset ownership) than in the richest quintile adolescents in Iran [70]. This was supported by a systematic review that showed that adolescents with low SES (income, housing, ownership of household durable assets, utilities) are at a higher risk of behavioural problems (e.g., low physical activity, consumption of high energy-dense food) [97], which is related to poorer HRQoL [98]. Higher burden of physical and mental illnesses and access barriers to care are other factors that explain impaired quality of life in the population with lower SES [99,100].

Ethnicity is among the factors that influence HRQoL and QALY, although the evidence is limited and inconsistent [101,102]. In this study, HRQoL and QALYs were lower in White ethnicity adolescents compared to ‘other ethnicity’ in DASH data. These findings are contrary to some studies among adolescents in the USA [101]. However, some studies in adolescent mental health in the UK found similar results with better mental health reported among ethnic minorities [43,103], although contrasting evidence is also available [104]. Inequalities in HRQoL to the disadvantage of ethnic minorities was also reported among older population in England [105]. There was no evidence of a significant association between ethnicity and HRQoL or QALYs in the HeadStart data. This may be explained by a lack of power to detect differences due to limited variability within ethnicity in the study sample, with ethnicity composition of 2.38% (non-White) and 97.62% (White).

In addition to the main effects analyses discussed above, this study included interaction analyses to further examine the moderation of the association of ACEs with HRQoL and QALYs by characteristics of the study population. A small body of literature shows that there are differences in the distribution of ACEs and in the effect of ACEs on population wellbeing. For instance, it is reported that ACEs are disproportionately prevalent among marginalised populations such as women, ethnic minorities, low SES, and less educated populations [3]. Furthermore, significant inequalities in ACEs scores across the intersections of sex and SES have been reported [106,107], with the highest ACE scores observed in multiracial female adolescents from low-income households, followed by low-income multiracial male adolescents (12–17 years of age) in the US [106]. This study also showed a higher prevalence of ACEs among non-White females and the poor (**Supplementary Table 9**).

While some studies assessed how patient characteristics moderate the link between ACEs and mental health [108,109,110], moderation analyses in HRQoL and QALYs are limited [29,30]. In this study, interaction term analyses generally produced inconsistent results, particularly in the follow-up surveys, which showed no statistically significant moderation. However, analysis of DASH data suggests a dose-dependent interaction between ACEs and ethnicity and gender (2+ ACEs) for the baseline HRQoL and QALYs. White respondents typically had lower HRQoL and QALYs, and this gap widened for those with a greater number of ACEs compared to all other ethnicity–ACE groups. Similarly, the dose-dependent ACEs-gender interaction showed a significant association, only for the interaction of gender with two and three or more counts of ACEs. However, none of the ACE-SES interactions was significant. Inconsistent moderation by gender and ethnicity, or the absence of moderation by SES, may suggest that ACEs were uniformly associated with HRQoL and/or QALYs across different sociodemographic characteristics. Although females and the poor were more likely to experience ACEs (**Supplementary Table 9**), once exposed, the detrimental effects of ACEs appeared consistent across all groups. Therefore, while targeted prevention policies can address unequal exposure to ACEs, universal policies, such as school-based trauma-informed care, are needed to mitigate the effects of ACEs on the exposed. Non-significant or inconsistent interaction effects do not necessarily imply a lack of moderation but may reflect methodological limitations, such as insufficient statistical power or measurement issues. This is an area of research that needs further investigation with higher-quality data and rigorous analytic methods.

### Limitations

The study findings have some limitations that should be considered. First, although the study used longitudinal adolescent surveys, there were differences in the ethnic profiles of respondents and the type and measures of ACEs and SES across the two datasets. While binary ethnicity was available in the HeadStart data, DASH data had multicategory ethnicity. We have collapsed the ethnicity categories in DASH data into two to align with the HeadStart data. While this categorisation simplifies analyses, we acknowledge that it may have introduced bias by masking variation in HRQoL and/or QALYs across different ethnic groups [111,112,113]. For instance, Lam [113] reported that, granular ethnicity categorisation identified meaningful differences and inequities in experiences of racism compared to coarse categories. Therefore, future studies may explore granular and intentional categorisation of race/ethnicity to assess its association with HRQoL/QALYs and whether the association between ACEs and HRQoL/QALYs is moderated by race/ethnicity. In terms of SES, the HeadStart data had a single variable indicating the presence or absence of ACEs based on official Supporting Families Program data, while the DASH data included eight categories of adversity based on self-report data. Different methods of data collection about ACEs are problematic because official reports usually under-estimate ACEs [4]. This is evident in the HeadStart data, which showed only 12% prevalence of ACEs, compared to 59% in the DASH data. Furthermore, the binary nature of the ACE variable in the HeadStart data limited the analysis of the potential graded effects of ACEs, which is shown in the DASH data. Second, HRQoL was predicted by mapping SDQ using a statistical mapping algorithm [49]; any prediction inaccuracy may have biased the results. Third, the data sources did not specify whether the variable sex represents biological sex or gender identity; we have assumed it represents gender, and this may have affected interpretations of study findings. These limitations notwithstanding, large sample sizes with varied population groups suggest the findings of the study may be generalisable to adolescents (11–17 years old) in the UK.

## 5. Conclusions

This study adds to a sparse literature base showing that the effects of ACEs extend beyond biomedical morbidity in young people, reducing subjective multidimensional health, as reflected in HRQoL and QALYs. Exposure to ACEs, both self-reported and government-recorded, is associated with lower HRQoL and greater QALY losses, with the strength of association increasing as the number of ACEs increases. Furthermore, adolescents with White ethnicity have relatively larger QALY losses compared to other ethnicities. Older female adolescents also have lower QALYs than their counterparts. These findings suggest that the effect of ACEs on HRQoL and QALYs differs by adolescents’ background characteristics, and warrant additional investigation of the pathways of effect, including more detailed and granular ethnic classifications and more robust, high-quality measures of SES, to inform policies that reduce inequalities. The findings also suggest better targeting of preventive interventions with universal mitigation interventions for ACEs and ACE-related health problems, guided by how impacts vary across different populations.

## Data Availability

The HeadStart data are available for secondary analysis through the UK Data Service. DASH data are available to researchers upon request to seeromanie.harding@kcl.ac.uk.
