# Supplementary material for: Association of Adverse Childhood Experiences With Health-Related Quality of Life and Quality-Adjusted Life-Years Among Adolescents in the United Kingdom: Findings From Two General Population Cohorts
Source: Alpha Psychiatry. 2026 Jun 29;27(3):49284. doi: 10.31083/AP49284 (PMC13339791; doi:10.31083/AP49284)
Supplement: Supplementary file 1 [file 2757-8038-27-3-49284-s1.zip › Supplementary Material.docx]

**Supplementary Materials**

**Supplementary Table 1. Types of self-reported adverse childhood experiences in DASH data**

| **ACEs** | **Wave 1** | | **Wave 2** | |
| --- | --- | --- | --- | --- |
|  | **N** | **%** | **N** | **%** |
| **Foster parents or children’s home** |  |  |  |  |
| Yes | 24 | 0.36 | 19 | 0.29 |
| No | 2,619 | 39.4 | 4,705 | 70.77 |
| **Parents alive?** |  |  |  |  |
| Mum and dad alive | 2,404 | 36.16 | 4,472 | 67.27 |
| Mum or dad or both not alive | 257 | 3.87 | 235 | 3.53 |
| **Parent/s has cancer** |  |  |  |  |
| Mum/dad or both has cancer | 58 | 0.87 | 82 | 1.23 |
| Both mum and dad do not have cancer | 4,124 | 62.03 | 3,881 | 58.38 |
| **Parent/s has mental illness** |  |  |  |  |
| Mum/dad or both has mental illness | 335 | 5.04 | 338 | 5.08 |
| Both mum and dad do not have mental illness | 3,624 | 54.51 | 3,486 | 52.44 |
| **Hassled due to race (home, school, street)** |  |  |  |  |
| Yes | 1,207 | 18.16 | 1,338 | 20.13 |
| No | 3,735 | 56.18 | 3,377 | 50.8 |
| **Hassled due to religion (home, school, street)** | |  |  |  |
| Yes | 486 | 7.31 | 475 | 7.15 |
| No | 4,429 | 66.62 | 4,239 | 63.76 |
| **Hassled due to looks/speech (home, school, street)** | |  |  |  |
| Yes | 1,768 | 26.59 |  |  |
| No | 3,136 | 47.17 |  |  |
| **Parents separated/divorced** |  |  |  |  |
| Yes | 1,822 | 27.41 | 1,675 | 25.2 |
| No | 1,115 | 16.77 | 3,044 | 45.79 |

**Supplementary Table 2. Variables used for generating wealth index in DASH data**

| **Variable** | **Yes/ No** |
| --- | --- |
| Car |  |
| CD / hi-fi CD player |  |
| Video or DVD player |  |
| Garage |  |
| Bedrooms |  |
| TV |  |
| Telephone |  |
| Home computer |  |
| Toilet |  |
| Holidays abroad each year |  |
| Freezer |  |
| Dishwasher |  |
| Garden |  |
| Washing machine |  |
| Microwave |  |
| Satellite/cable |  |
| Tumble dryer |  |
| Own bedroom |  |
| Own pc |  |
| Own mobile |  |
| Access to internet |  |
| Pocket money |  |
| Money for jobs |  |
| Weekly money received |  |

**Supplementary Table 3. Predictors of drop out at the follow-up survey of DASH and HeadStart**

|  | **DASH** | | | **HeadStart** | | |
| --- | --- | --- | --- | --- | --- | --- |
|  | **Odds Ratio** | **95% CI** | | **Odds Ratio** | **95% CI** | |
| Baseline total SDQ | 1.02 | [1.01 | 1.03] | 1.00 | [0.99 | 1.01] |
| ACE |  |  |  |  |  |  |
| Administrative data |  |  |  | 1.69 | [1.39 | 2.06] |
| Self-report | 0.99 | [0.88 | 1.11] |  |  |  |
| Female | 1.24 | [1.11 | 1.39] | 0.98 | [0.86 | 1.11] |
| White | 1.27 | [1.13 | 1.43] | 0.91 | [0.63 | 1.31] |
| Non poor | 0.87 | [0.72 | 1.05] | 1.06 | [0.87 | 1.28] |
| Constant | 0.29 | [0.23 | 0.37] | 0.48 | [0.31 | 0.76] |

**Supplementary Table 4. Number of missing values imputed for each Variable**

|  | **DASH (N=6818)** | | | | **HeadStart (N=4575)** | | | |
| --- | --- | --- | --- | --- | --- | --- | --- | --- |
| **Variable** | **2002/03** | | **2004/05** | | **2017** | | **2019** | |
|  | **No.** | **%** | **No.** | **%** | **No.** | **%** | **No.** | **%** |
| ACEs | 371 | 5.44 | 1894 | 27.78 | 0 | 0.00 | 0 | 0.00 |
| Gender | 22 | 0.32 |  |  | 2 | 0.04 |  |  |
| Socioeconomic status | 411 | 6.03 |  |  | 0 | 0.00 |  |  |
| Ethnicity | 17 | 0.25 |  |  | 69 | 1.51 |  |  |
| I try to be nice to other people. I care about their feelings | 122 | 1.79 | 1881 | 27.59 | 133 | 2.91 | 1563 | 34.16 |
| I am restless, I cannot stay still for long | 157 | 2.30 | 1884 | 27.63 | 133 | 2.91 | 1568 | 34.27 |
| I get a lot of headaches, stomach-aches or sickness | 165 | 2.42 | 1892 | 27.75 | 164 | 3.58 | 1585 | 34.64 |
| I usually share with others (food, games, pens etc.) | 172 | 2.52 | 1880 | 27.57 | 149 | 3.26 | 1577 | 34.47 |
| I get very angry and often lose my temper | 145 | 2.13 | 1882 | 27.60 | 145 | 3.17 | 1575 | 34.43 |
| I am usually on my own. I generally play alone or keep to myself | 151 | 2.21 | 1884 | 27.63 | 136 | 2.97 | 1570 | 34.32 |
| I usually do as I am told | 142 | 2.08 | 1877 | 27.53 | 142 | 3.10 | 1570 | 34.32 |
| I worry a lot | 143 | 2.10 | 1877 | 27.53 | 140 | 3.06 | 1571 | 34.34 |
| I am helpful if someone is hurt, upset or feeling ill | 156 | 2.29 | 1884 | 27.63 | 154 | 3.37 | 1578 | 34.49 |
| I am constantly fidgeting or squirming | 210 | 3.08 | 1902 | 27.90 | 139 | 3.04 | 1570 | 34.32 |
| I have one good friend or more | 173 | 2.54 | 1889 | 27.71 | 163 | 3.56 | 1575 | 34.43 |
| I fight a lot. I can make other people do what I want | 161 | 2.36 | 1885 | 27.65 | 145 | 3.17 | 1572 | 34.36 |
| I am often unhappy, downhearted or tearful | 176 | 2.58 | 1888 | 27.69 | 163 | 3.56 | 1579 | 34.51 |
| other people my age generally like me | 178 | 2.61 | 1889 | 27.71 | 157 | 3.43 | 1572 | 34.36 |
| I am easily distracted; I find it difficult to concentrate | 183 | 2.68 | 1887 | 27.68 | 166 | 3.63 | 1572 | 34.36 |
| I am nervous in new situations I easily lose confidence | 168 | 2.46 | 1888 | 27.69 | 151 | 3.30 | 1569 | 34.30 |
| I am kind to younger children | 162 | 2.38 | 1884 | 27.63 | 160 | 3.50 | 1579 | 34.51 |
| I am often accused of lying or cheating | 161 | 2.36 | 1893 | 27.76 | 167 | 3.65 | 1579 | 34.51 |
| Other children or young people pick on me or bully me | 197 | 2.89 | 1883 | 27.62 | 163 | 3.56 | 1581 | 34.56 |
| I often volunteer to help others (parents, teachers, children) | 212 | 3.11 | 1891 | 27.74 | 154 | 3.37 | 1574 | 34.40 |
| I think before I do things | 208 | 3.05 | 1887 | 27.68 | 171 | 3.74 | 1577 | 34.47 |
| I take things that are not mine from home, school or elsewhere | 209 | 3.07 | 1888 | 27.69 | 164 | 3.58 | 1570 | 34.32 |
| I get on better with adults than with people my own age | 221 | 3.24 | 1892 | 27.75 | 165 | 3.61 | 1568 | 34.27 |
| I have many fears; I am easily scared | 220 | 3.23 | 1883 | 27.62 | 170 | 3.72 | 1585 | 34.64 |
| I finish the work I'm doing. My attention is good | 212 | 3.11 | 1881 | 27.59 | 174 | 3.80 | 1577 | 34.47 |

**Supplementary Table 5. Multiple imputation robustness assessment using Monte Carlo errors of regression of follow up predicted utilities.**

|  | **DASH** | | | **HeadStart** | | |
| --- | --- | --- | --- | --- | --- | --- |
|  | **Coefficient**  **[MCE errors]** | **SE**  **[MCE errors]** | **Relative MCE (MCE of coefficient / SE)** | **Coefficient**  **[MCE errors]** | **SE**  **[MCE errors]** | **Relative MCE (MCE of coefficient / SE)** |
| ACEs |  |  |  |  |  |  |
| Self-report | -0.030 | 0.009 |  |  |  |  |
|  | [0.001] | [0.000] | 0.077 |  |  |  |
| Administrative data |  |  |  | -0.015 | 0.014 |  |
|  |  |  |  | [0.001] | [0.000] | 0.076 |
| Female | -0.075 | 0.004 |  | -0.093 | 0.005 |  |
|  | [0.000] | [0.000] | 0.074 | [0.000] | [0.000] | 0.074 |
| Non poor | 0.009 | 0.008 |  | 0.031 | 0.009 |  |
|  | [0.001] | [0.000] | 0.081 | [0.001] | [0.000] | 0.068 |
| White | -0.010 | 0.003 |  | -0.021 | 0.013 |  |
|  | [0.000] | [0.000] | 0.066 | [0.001] | [0.000] | 0.064 |
| ACEs and female | 0.002 | 0.005 |  | -0.038 | 0.014 |  |
|  | [0.000] | [0.000] | 0.070 | [0.001] | [0.000] | 0.078 |
| ACEs and non-poor | 0.005 | 0.010 |  | -0.025 | 0.015 |  |
|  | [0.001] | [0.000] | 0.079 | [0.001] | [0.000] | 0.070 |
| Constant | 0.790 | 0.008 |  | 0.757 | 0.015 |  |
|  | [0.001] | [0.000] | 0.077 | [0.001] | [0.000] | 0.068 |

**Notes:**  MCE – Monte Carlo errors of estimates and standard errors, in square brackets, indicate variability of estimates across repeated imputations. Relative MCE – MCE of the parameters relative to the SE. Less than 0.10 relative MCE indicates sufficient number of imputations.

**Supplementary Fig. 1. Distribution of predicted utilities in DASH and HeadStart baseline and follow up**

**DASH – predicted utilities for imputation no. 1**

**HeadStart**

**Supplementary Table 6. Multiple imputation estimates of pooled mean of predicted utilities across SDQ quintiles**

|  |  | **Baseline** | | **Follow-up** | |
| --- | --- | --- | --- | --- | --- |
|  |  | **Mean** | **SE** | **Mean** | **SE** |
| Quintiles of total SDQ - DASH | 1 | 0.870 | 0.000 | 0.823 | 0.000 |
|  | 2 | 0.840 | 0.000 | 0.787 | 0.000 |
|  | 3 | 0.799 | 0.000 | 0.754 | 0.000 |
|  | 4 | 0.737 | 0.001 | 0.717 | 0.000 |
|  | 5 | 0.654 | 0.001 | 0.631 | 0.001 |
| **Overall** | | **0.791** | **0.001** | **0.746** | **0.001** |
|  |  |  |  |  |  |
| Quintiles of total SDQ - HeadStart | 1 | 0.864 | 0.000 | 0.842 | 0.000 |
|  | 2 | 0.806 | 0.001 | 0.777 | 0.001 |
|  | 3 | 0.750 | 0.000 | 0.726 | 0.001 |
|  | 4 | 0.682 | 0.001 | 0.644 | 0.001 |
|  | 5 | 0.577 | 0.001 | 0.526 | 0.001 |
| **Overall** | | **0.743** | **0.002** | **0.712** | **0.002** |

**Supplementary Table 7. Multiple imputation bivariate regression of predicted utilities on total SDQ**

|  | **Dependent** | **Predictor** | **Estimate** | **SE** |
| --- | --- | --- | --- | --- |
| DASH | Utility baseline | Baseline total SDQ | -0.016 | 0.000 |
|  | Utility follow-up | Follow-up total SDQ | -0.015 | 0.000 |
| HeadStart | Utility baseline | Baseline total SDQ | -0.020 | 0.000 |
|  | Utility follow-up | Follow-up total SDQ | -0.015 | 0.000 |

**Supplementary Table 8. Multiple imputation estimates of predictors of total SDQ score in DASH and HeadStart respondents.**

|  | **DASH** | | | | **HeadStart** | | | |
| --- | --- | --- | --- | --- | --- | --- | --- | --- |
|  | **Baseline SDQ** | | **Follow-up SDQ** | | **Baseline SDQ** | | **Follow-up SDQ** | |
|  | **Estimate** | **SE** | **Estimate** | **SE** | **Estimate** | **SE** | **Estimate** | **SE** |
| **Main effects** |  |  |  |  |  |  |  |  |
| ACEs |  |  |  |  |  |  |  |  |
| Self-report | 1.214 | 0.412*** | 1.051 | 0.460** |  |  |  |  |
| Administrative data |  |  |  |  | 1.817 | 0.446*** | 0.385 | 0.535 |
| Gender | 0.645 | 0.179*** | 0.623 | 0.193*** | 0.475 | 0.158*** | 1.842 | 0.185*** |
| Non poor | -0.552 | 0.328* | -0.257 | 0.36 | -0.848 | 0.278*** | -0.714 | 0.328** |
| White | 0.357 | 0.123*** | 0.427 | 0.124*** | 0.459 | 0.432 | 0.234 | 0.535 |
| **Interactions** |  |  |  |  |  |  |  |  |
| ACEs and female | 0.14 | 0.234 | 0.208 | 0.256 | -0.071 | 0.438 | 0.909 | 0.513* |
| ACEs and non-poor | -0.071 | 0.421 | -0.563 | 0.464 | -0.214 | 0.493 | 0.858 | 0.576 |
| Constant | 14.36 | 0.322*** | 14.415 | 0.357*** | 17.03 | 0.506*** | 16.531 | 0.612*** |

**Supplementary Table 9. Prevalence of ACEs by gender, ethnicity and socioeconomic status intersectionality profile of respondents at baseline**

|  | **Intersectionality Profile** | | **Intersectionality Profile reporting ACEs** | | **Intersectionality Profile** | | **Intersectionality Profile reporting ACEs** | |
| --- | --- | --- | --- | --- | --- | --- | --- | --- |
|  | **DASH** | | | | **HeadStart** | | | |
|  | **N** | **%** | **N** | **%** | **N** | **%** | **N** | **%** |
| White non poor | 1,716 | 27.52 | 1,023 | 59.62 | 3,703 | 87.96 | 305 | 8.24 |
| White poor | 169 | 2.71 | 103 | 60.95 | 407 | 9.67 | 201 | 49.39 |
| Non-White non poor | 3,896 | 62.48 | 2,255 | 57.88 | 83 | 1.97 | 7 | 8.43 |
| Non-White poor | 455 | 7.3 | 274 | 60.22 | 17 | 0.4 | 12 | 70.59 |
| **Total** | **6,236** | **100** | **3,655** | **58.61** | **4,210** | **100** | **525** | **12.47** |
|  |  |  |  |  |  |  |  |  |
| Female non poor | 2,650 | 42.52 | 1,616 | 60.98 | 1,856 | 43.69 | 153 | 8.24 |
| Female poor | 292 | 4.68 | 188 | 64.38 | 224 | 5.27 | 116 | 51.79 |
| Male non poor | 2,960 | 47.49 | 1,662 | 56.15 | 1,967 | 46.3 | 161 | 8.19 |
| Male poor | 331 | 5.31 | 189 | 57.10 | 201 | 4.73 | 96 | 47.76 |
| **Total** | **6,233** | **100** | **3,655** | **58.64** | **4,248** | **100** | **526** | **12.38** |
|  |  |  |  |  |  |  |  |  |
| Female White | 908 | 13.7 | 561 | 61.78 | 1,995 | 47.61 | 255 | 12.78 |
| Female non-White | 2,183 | 32.95 | 1,271 | 58.22 | 54 | 1.29 | 12 | 22.22 |
| Male White | 1,053 | 15.89 | 575 | 54.61 | 2,096 | 50.02 | 246 | 11.74 |
| Male non-White | 2,482 | 37.46 | 1,311 | 52.82 | 45 | 1.07 | 7 | 15.56 |
| **Total** | **6,626** | **100** | **3,718** | **56.11** | **4,190** | **100** | **520** | **12.41** |
